# Miscellaneous Observations, in the Manner of Axioms, Respecting Small-Pox

**Published:** 1816-07

**Authors:** Richard Walker

**Affiliations:** of Oxford.


*For the London Medical and Physical Journal.*

*Miscellaneous Observations, in the manner of Axioms, respecting Small-pox;* by RICHARD WALKER, Esq. of Oxford.

SECTION I.—A person receiving small-pox casually from another, (that is, in the natural way,) sickens at the same distance of time nearly, but probably somewhat later than when communicated by inoculation, since it is a common custom to introduce into the very room of the person, however virulent that person may have the disease; notwithstanding which, the affection in all instances is the effect alone of the inoculation; but it is uncertain when, or at what period exactly, a person living in the same house may take the infection of small-pox from another; it is certain, however, (an unquestionable instance of which I have witnessed,) that a person may take the infection from another by the breath, during the eruptive indisposition.

Sect. II.—A person, before the seizure of the more evident variolous symptoms preceding small-pox, is not unfrequently slightly indisposed for a few days before, with lassitude, drowsiness, or heaviness, and has less inclination than usual to his meals; but this is ordinarily so slight as to be overlooked at the time.

Sect. III.—In the distinct kind of small-pox, the eruptive fever is synocha. In the confluent at first synocha, changing in the course of the disease to typhus; and sometimes typhus at the very commencement.

Sect. IV.—The degree of safety or danger respecting small-pox, depends principally, or almost exclusively, upon the quantity and nature of the eruption upon the face, without regard to the body or limbs.

Sect. V.—The pustules in the distinct small-pox are circular, and become considerably elevated; in the confluent, the pustules are not circular, but irregular at their base, even where separate, or not running one into another, are flatter or much less elevated; and the vesicle at the apex or centre appears earlier. Moreover there may be observed a regular gradation in the nature or kind of pustules, from the truly truly distinct kind of pustule, to the truly confluent kind, which may be divided into a series of four well-defined gradations, viz. 1st. the pustule with the perfectly circular base, which at the time of maturity becomes elevated to a perfectly hemispherical figure; 2dly, the pustule with an irregular and more extended base, flat or much less elevated, but still a single pustule; 3dly, the pustule possessing nearly the same character as the latter, but which is composed of two or more pustules running into one, or joined together rather, forming a smaller or greater degree of confluence; and, 4thly, the truly confluent kind, in which the pustules are united into complete patches of various extent.

Sect. VI.—In the distinct kind, especially when the pustules are numerous, and become pretty large or near maturity, the interstices between them, formed by a junction, as it were, of the inflamed bases of the whole collectively, is of a bright red or damask rose colour, especially in the face; in the truly confluent kind, or the approximation to it, the interstices are less vivid, and in the worst kind, quite pallid.

Sect. VII.—In the distinct kind, unless the patient is pretty much loaded, the fever and indisposition ceases when the eruption is completed, and returns no more, or very slightly, at the time of maturation, viz. about the ninth or tenth day; but in the confluent kind, especially in the more crowded or virulent sort, the fever scarcely remits or abates after the eruption is completed, becoming more violent at the time of maturation.

Sect. VIII.—The progress of the pustules is ordinarily thus: they appear first in the face, neck, and breast; next in the body and upper limbs; and lastly the lower limbs; after the same order they come to maturity; but slower, after the same order, in the hands and feet for instance, in reaching maturity, reckoning from the time of their coming out. In the confluent kind, I have observed pustules at their height in the feet, so late as the twenty-first or twenty-second day, which were the last remaining.

In the hands and feet, particularly in the confluent kind, the pustules, or vesicles rather, toward the end, become empty, the fluid having either been absorbed or transuded; and this occurs without any indication of danger.

In the mildest cases of the distinct kind, the pustules sometimes, even in the face, do not arrive at a purulent maturation, but ripen and dry off without; this circumstance, in persons not thoroughly conversant in small-pox, sometimes creates a doubt respecting the nature of the complaint. The same circumstance occasionally occurs in inoculation, even when when the matter has been taken from the most virulent kind of small-pox.

Sect. IX.—The truly distinct small-pox, however full or loaded the patient may be, ordinarily terminates well. In the truly confluent small-pox, according to my observation, when it proves fatal, the patient dies more commonly on the fourteenth or fifteenth day, but sometimes earlier or later; if, however, the patient pass the latter of those days with an amendment, or without an aggravation of the symptoms, a favourable event may be confidently hoped for. The circumstances which more particularly indicate danger about, or anterior to, the critical days above-mentioned, are a great increase of hoarseness, so as nearly to destroy the voice; the phlegm, or matter of salivation, becoming so viscid as to be expectorated with extreme difficulty; violent delirium, or coma; a pallid shrunk countenance; feeble quick pulse; shortness of breath, &c. The more immediate precursors of death in this disease, as in other fevers, &c. viz. pinching and gathering up the bed-clothes, and a greater or lesser degree of stertor, quickly follow the former bad symptoms, and the patient expires; though it sometimes happens that the patient dies rather suddenly, without the occurrence of the more alarming train of symptoms.

Sect. X.—In the worst kind of confluent small-pox, the pustules, or rather patches of pustules in the forehead, remains pallid and not matured to the fourteenth or fifteenth day; such persons seldom recover.

Sect. XI.—For the most part, and for a very obvious reason, the small-pox is, *cæteris paribus*, more virulent, and consequently more dangerous, in hot than in cool, or cold, weather; and, moreover, the patient is less able then to sustain himself at the crisis.*

---

* In very hot weather, the secondary fever, and other annoying circumstances, as the local irritation of the pustules, &c. are very much increased; hence every means should be exerted to obtain a cool temperature; this may be effected to a very considerable degree by evaporation judiciously managed; but much more powerfully and uniformly, if conveniently practicable, by a refrigeratory placed in the room, during the most critical period, consisting of ice, or a mixture of ice and salt; this, as I well know by experience, will lower the temperature of a room very considerably. The same observation applies equally in cases of scarlatina and typhus. Cold weather is unfavourable to the propagation of small-pox; hence, it happens that small-pox ordinarily appears first in the spring, becomes more general and violent during summer, abates in the autumn, and disappears, or becomes much less prevalent, during the winter.

Sect. XII.—Petechiæ, or the purples as they are sometimes called, although an untoward or alarming circumstance, according to the degree of putrescency, do not constantly prognosticate a fatal termination.

Sect. XIII.—An erythematic kind of rash, in the face more particularly, sometimes precedes the confluent kind of small-pox, the variolous eruption seeming to emerge from this rash in minute red spots somewhat elevated, resembling meazles, for which it might be at first mistaken by an unwary practitioner, where small-pox was not expected. The same circumstance happens likewise in the distinct kind, even where the affection turns out of a perfectly favourable kind; I have witnessed it in inoculation, threatening to the inexperienced, a very full eruption, but not thus followed; likewise, in small-pox taken casually after vaccination, where, as is ordinarily the case in such instances, the affection was of the mildest kind.*

Sect. XIV.—In the confluent or more virulent kind of small-pox, every attendant circumstance is not only more violent, but of longer continuance; thus, the swelling of the face begins earlier, rises to a greater height and continues longer; and the same, likewise, respecting the spitting or ptyalism; and the blindness lasts longer, viz. for a week or more, notwithstanding which, the patient may recover.

Sect. XV.—It is observed, that a disposition to sweating, previous and subsequent to the eruption, is a favourable circumstance, indicating a mild affection; and a disposition to diarrhœa contrariwise. This may be probably accounted for by the variolous matter being carried off more effectually or copiously, and with less exhaustion of the habit, by the former than by the latter excretion; hence it might be inferred, that, if the pores of the skin were rendered open by art, without increasing the heat, we might effect the same consequence;

---

* The variolous eruption, in all these instances, whether it be of the distinct or confluent kind, is quickly distinguishable by the vesicular appearance in the apex or centre of those eruptions, whilst the erythematic appearance fades and vanishes.

I have noticed in several children who have passed through vaccination, and mostly so, I think, in such as are of a gross or full habit, small incysted tumours about the size of warts, sometimes single and sometimes in clusters; these, after some time, inflame, burst, and ultimately disappear; they come chiefly in the face or neck, and, if single, may be taken out. Whether this is the effect of vaccination, I do not know, but three children, within my own knowledge, have them at this time; but, as they ordinarily go away spontaneously, it is a matter of no importance.

consequence; but this practice would be extremely precarious, and we are in possession of a much better method, viz. moderating the degree of heat, and thereby preventing so large an accumulation, or assimilation of the variolous matter to be thrown off.

Sect. XVI.—In the confluent small-pox, the pustules run more together in the face than elsewhere, and the single or separate pustules are larger [more extended] on the hands and feet, than those on other parts, and become gradually less as they approach the body.

Sect. XVII.—The eruptions in the distinct small-pox are, at their first appearance, red circular spots, scarcely elevated, and of a sensible diameter, and detached from each other; whereas the eruptions in the confluent kind, are at first mere red points, crowded less or more in clusters, which afterwards coalesce into one; so that even those which appear as separate pustules, are composed of two or more of these points coalesced together, which occasions the irregular base, or margin, and flatness, even in those apparently separate pustules.

Sect. XVIII.—It sometimes unfortunately happens that one or more pustules form upon the cornea, exactly before the pupil, which occasions an incurable blindness; this circumstance occurring ordinarily during the time the eyes are closed by the tumefaction of the eye-lids, does not afford an opportunity of its being obviated by art. A blister to the nape of the neck, where such a circumstance is apprehended, might prevent it.

Sect. XIX.—Pitting, as I apprehend, occurs in consequence of a succession of crusts, or scabs, formed in the course of the ulcerated pustules healing naturally, and which could only be prevented by healing them *secundum artem*, as in other instances of sores, without suffering any scab to form, to leave an impression; such pits are constantly the effect, and from the same cause in the part inoculated or vaccinated; and this effect is not unfrequently increased by the patients, particularly children, picking them. The matter of the pustules in small-pox is, in different instances, of a lesser or greater degree of virulence, or acrimonious, or corroding nature; in proportion to this will be the degree of pitting, especially when suffered to form crusts or scabs; and which, from the multiplicity of them, cannot easily be prevented by art.

Sect. XX.—There are several anomalous varieties of small-pox, which occasionally occur in practice; viz. the crystalline, in which the fluid never becomes opake or purulent; the vesicular, in which small vesicles appear in the interstices terstices of the pustules, &c., but which are all merely different modifications of the same disease; and likewise, occasionally, other deviations from the ordinary course, or progress of small-pox, both in the distinct and confluent kind: but my intention here being to exhibit a journal of the ordinary appearances and progress of small-pox; and as these anomalies, accordingly as they approximate to one or other of the kinds of small-pox already described, require a similar mode of treatment, I have no need of noticing them.

Sect. XXI.—I have never been able to ascertain, by my own observation, nor can I meet with any satisfactory account respecting it, whether the small-pox ever occurs spontaneously, that is, without being propagated by infection in some way; nor whether the essence of the disease exists latently in the habit until excited to action; or that there merely exists in the habit a disposition to produce this disease, which can be effected only by receiving the essence of the disease by contagion. I am inclined to the former opinion, first, because it is difficult to account otherwise for its origin; and, secondly, because when the habit is once cleared of it, it is never afterwards ordinarily susceptible of it.

Sect. XXII.—The most confluent or virulent kind of small-pox, far more commonly than the distinct or milder sort, is followed by, or leaves behind it, more serious constitutional affections, as scrofula, &c. as I have frequently witnessed, which is an additional and very strong reason for securing a mild affection by inoculation or vaccination, whichever may be preferred.

*May*, 1816.

